# From Immunomonitoring to Immune Intervention

**DOI:** 10.3389/fimmu.2014.00669

**Published:** 2015-01-06

**Authors:** Antoine Toubert, Hermann Einsele

**Affiliations:** ^1^Immunology-Histocompatibility, Laboratoire d’Immunologie et d’Histocompatibilité, Laboratoire Jean Dausset, INSERM, Assistance Publique Hôpitaux de Paris, Université Paris Diderot, Paris, France; ^2^Department of Internal Medicine II, University Hospital Würzburg, Würzburg, Germany

**Keywords:** HSCT, immune reconstitution, thymic function, cell therapy, Haplo-SCT

Host immune status is a key issue in allogeneic hematopoietic stem cell transplantation (allo-HSCT). In the long-term follow-up of these patients, severe post-transplant infections, relapse or secondary malignancies may be directly related to prolonged immune defects, especially in the context of innovative stem cell sources such as umbilical cord blood transplantation (CBT) (1) or transplants from HLA haplo-identical family donors (Haplo-HSCT) (2, 3).

This *Frontiers* Research Topic provides insights into mechanisms of immune reconstitution in an allogeneic environment, in order to improve monitoring and therapeutic intervention in allo-HSCT patients.

Forcina et al. (4) critically review T and NK biomarkers, their threshold and clinical relevance. Many factors from the host, transplant conditioning, stem cell source, and genetic disparity may impact immune recovery. Few biomarkers have reached a clinical consensus: a rapid and potent CD4 T-cell recovery is associated with a favorable clinical outcome, CMV-specific CD8 T-cell counts measured by tetramers can predict a lower incidence of CMV disease. Other phenotypic or molecular markers, still based on small-sized studies, will need further validation in large scale-multicentric cohorts. This is the case of molecular markers of lymphocyte generation, such as T-cells evaluated by the quantification of T-cell rearrangement excision circles (TREC) as a surrogate marker of thymic activity. Clave et al. (3) indicate an impact of thymic function recovery on relapse following CBT as well as Haplo-HSCT in children treated for an hematological malignancy. In order to reach the routine medical practice, immunomonitoring tests need also to be simple and fast. This practical issue is well taken into account by Fuji et al. (5) reviewing T-cell monitoring of viral and fungal infections. CMV is still the most intensively studied virus in immunocompromised hosts. CMV shapes T and NK cell responses in many ways and may escape immune response. Among still unanswered questions, is the role of viral-specific (CMV and EBV) T-cell cross-reactivity against allogeneic targets in graft-versus-host and graft-versus-leukemia (6).

Experimental models have proved their importance in allo-HSCT, especially deciphering mechanisms of allogeneicity in graft-versus-host disease (GVHD). Among rodents, rat models are relevant especially in some autoimmune conditions and solid organ transplantation, sometimes closer to the human disease than the “gold standard” murine models. Zinöcker et al. (7) emphasize the interest of rat model in experimental GVHD, especially the rat skin explant assay as a tool for functional evaluation of GVHD. Experimental models are also required to provide the basis and for preclinical evaluation of immune-based therapies.

This *Frontiers* Research Topic also reviews in some strategies already proposed in the clinics. Adoptive T-cell therapies have been conducted successfully by several groups to control life-threatening viral (EBV, CMV) reactivations. This leads to “off the shelf” strategies based on third party HLA-typed T-cell donors registries, which may have a major impact in transplant recipients (8). The development of therapeutic antibodies in cancer is one of the most active fields in clinical immunology. Many strategies are underway to improve their action, especially through their antibody-dependent cellular toxicity mediated by natural killer (NK) cells (9). One approach especially relevant in allo-HSCT would be to take advantage of the killer-immunoglobulin-like receptor (KIR) ligand incompatibility in addition to ADCC to boost NK functions.

The impact of these different concepts, from basic knowledge to translational medicine, is integrated in state-of-the art reviews of two main “success stories” in allo-HSCT: CBT (1) and Haplo-HSCT (2).

## Conflict of Interest Statement

The authors declare that the research was conducted in the absence of any commercial or financial relationships that could be construed as a potential conflict of interest.
